# Ocular Manifestations of Sickle Cell Disease With an Emphasis on Retinal Involvement

**DOI:** 10.7759/cureus.91018

**Published:** 2025-08-26

**Authors:** Markandeya Singh, Jawahar Jyoti Kuli, Anupam Dutta, Arpita Gogoi

**Affiliations:** 1 Department of Ophthalmology, Assam Medical College and Hospital, Dibrugarh, IND; 2 Department of General Medicine, Assam Medical College and Hospital, Dibrugarh, IND; 3 Department of Pediatrics, Assam Medical College and Hospital, Dibrugarh, IND

**Keywords:** northeast india, ocular manifestations, retinal involvement, sickle cell disease (scd), sickle cell retinopathy

## Abstract

Background

This study aimed to evaluate the spectrum of ocular manifestations in sickle cell disease (SCD) patients, with a particular emphasis on retinal involvement, to improve early diagnosis and management strategies.

Methodology

A cross-sectional, observational study was conducted over one year at Assam Medical College and Hospital, Dibrugarh, recruiting 96 SCD patients aged 10 and above. Comprehensive ophthalmological evaluations included best-corrected visual acuity, slit-lamp biomicroscopy, direct/indirect ophthalmoscopy, spectral-domain optical coherence tomography (OCT), and, in selected cases, fundus fluorescein angiography. Sickle cell retinopathy (SCR) was graded using Goldberg’s classification.

Results

The mean age of the participants was 31.00 ± 13.89 years, with 52 (54.17%) males and 71 (76.04%) from the tea tribe community. Systemic features included pallor in 85 (88.54%) and generalized weakness in 83 (86.46%) participants. Visual acuity of 6/6 to 6/9 was found in 32 (33.33%) (right eye) and 33 (34.38%) (left eye) participants. Anterior segment involvement (corkscrew vessels) was noted in 50 (52.08%) participants, and posterior segment changes were noted in 51 (53.13%) participants. Non-proliferative SCR was present in 46 (47.92%) and proliferative SCR in 5 (5.21%) participants. OCT revealed vitreomacular traction, epiretinal membranes, and full-thickness macular holes. Patient age correlated with retinopathy severity (R² = 0.76, p < 0.01), and systemic severity correlated with retinopathy (R² = 0.94, p < 0.001).

Conclusions

SCD patients exhibit significant ocular manifestations, particularly retinal pathology, which correlates with age and systemic disease severity. Early and routine ophthalmologic screening is crucial for timely management and preventing vision loss.

## Introduction

Sickle cell disease (SCD) is an inherited hemoglobinopathy caused by a point mutation in the β-globin gene, resulting in the production of abnormal hemoglobin S (HbS) [1]. This mutation leads to erythrocyte deformation, promoting hemolysis and vaso-occlusion, which contribute to a range of systemic and ocular complications [1]. While SCD is primarily associated with hematologic and systemic manifestations, its ocular implications, particularly on the retina, are significant yet often underrecognized [2].

Ocular manifestations of SCD encompass both anterior and posterior segment changes. Anterior segment findings include conjunctival vascular abnormalities, such as “corkscrew” vessels, and iris atrophy [3]. Posterior segment involvement is more prevalent and clinically significant, with sickle cell retinopathy (SCR) being a primary concern [3]. SCR is categorized into non-proliferative and proliferative forms, with the latter posing a substantial risk of vision loss due to neovascularization, vitreous hemorrhage, and retinal detachment [4]. The pathogenesis of these retinal changes is rooted in the occlusion of small retinal vessels, leading to ischemia, infarction, and subsequent neovascularization [4]. Goldberg’s classification further delineates the progression of proliferative sickle retinopathy (PSR) into five stages, emphasizing the importance of early detection and intervention [3].

Interestingly, PSR occurs more frequently in patients with HbSC and HbS/β-thalassemia genotypes compared to those with HbSS, despite the latter being associated with more severe systemic disease [5]. Studies have reported that PSR is present in up to 87.6% of HbSC individuals with retinopathy, compared to 60% in HbSS patients [6]. This paradox underscores the necessity of genotype-specific screening protocols [7]. Furthermore, the prevalence of ocular complications increases with age, highlighting the importance of regular ophthalmologic evaluations [7].

Advancements in retinal imaging techniques, such as wide-field fluorescein angiography and optical coherence tomography (OCT), have enhanced the detection of subclinical retinal changes in SCD patients [8]. These modalities facilitate the identification of early ischemic alterations and neovascularization, enabling timely management to prevent vision-threatening complications [8]. Recent studies utilizing OCT angiography have identified macular thinning and capillary dropout as early markers of retinal ischemia in SCD, even before clinical retinopathy becomes apparent [9].

Given the high prevalence of SCD in parts of India, Africa, and the Caribbean, implementing routine ophthalmologic screening from an early age is crucial [10]. Early identification and management of ocular complications can significantly reduce the risk of permanent visual impairment in affected individuals.

This study aims to evaluate the spectrum of ocular manifestations in SCD patients, with a particular emphasis on retinal involvement, to improve early diagnosis and management strategies. The research question guiding this study is: “What are the ocular manifestations of sickle cell disease, with a special emphasis on the retina?” By addressing this question, we hope to enhance the understanding of retinal complications in SCD and contribute to the development of targeted screening protocols.

## Materials and methods

This cross-sectional observational study was conducted over a one-year period at the Department of Ophthalmology, Assam Medical College and Hospital, Dibrugarh, with the aim of delineating the spectrum of ocular manifestations in patients with SCD, with a particular emphasis on retinal pathology. A total of 96 patients with confirmed diagnoses of SCD were prospectively recruited from the Departments of Ophthalmology, Medicine, and Pediatrics. Written informed consent was obtained from all participants or their legal guardians, in accordance with institutional guidelines.

Eligibility criteria

Inclusion criteria comprised individuals aged 10 years and above with laboratory-confirmed SCD by hemoglobin electrophoresis, who consented to undergo a comprehensive ophthalmological evaluation. Patients were excluded if they had pre-existing ocular pathologies unrelated to SCD (e.g., diabetic retinopathy, hypertensive retinopathy), had a history of ocular trauma or intraocular surgery, were receiving systemic medications known to affect ocular physiology, or were unwilling or unable to participate.

Clinical and ophthalmic evaluation

A structured clinical evaluation was performed, capturing demographic variables, relevant systemic and ocular history, and prior SCD-related complications. Best-corrected visual acuity (BCVA) was assessed using a standard Snellen chart under uniform illumination. Anterior segment evaluation was conducted using a Haag-Streit BQ 900 slit-lamp biomicroscope (Haag-Streit AG, Switzerland), enabling detailed assessment of conjunctival vasculature, corneal clarity, iris structure, and anterior chamber reactions. Intraocular pressure was measured using a Goldmann applanation tonometer (Haag-Streit AT 900, Switzerland).

Posterior segment assessment included both direct and indirect ophthalmoscopy. Central retinal features were evaluated using a Welch Allyn direct ophthalmoscope (Welch Allyn, USA), while peripheral retinal changes were examined using a Keeler Vantage Plus binocular indirect ophthalmoscope (Keeler Instruments, UK) in conjunction with a 20 D Volk lens. High-resolution retinal imaging was performed using spectral-domain OCT with the Heidelberg Spectralis system (Heidelberg Engineering, Germany), allowing for the quantification of macular thickness and integrity of the retinal nerve fiber layer. In selected cases, fundus fluorescein angiography (FFA) was performed using the Zeiss Visucam 500 (Carl Zeiss Meditec, Germany) to detect capillary non-perfusion, vascular occlusions, and areas of neovascularization.

Classification and statistical analysis

The severity of SCR was graded according to Goldberg’s classification system, which stratifies retinopathy into five progressive stages [5]. All clinical data were documented using a standardized proforma.

Data analysis was performed using descriptive statistics, including means, standard deviations (SDs), and percentage distributions. Categorical variables were analyzed using the chi-square (χ²) test, and a p-value <0.05 was considered statistically significant. Statistical computations were performed using SPSS Statistics version 25.0 (IBM Corp., Armonk, NY, USA) and Microsoft Excel 2010 (Microsoft Corp., Redmond, WA, USA).

The sample size of 96 was determined based on the estimated prevalence of ocular involvement in SCD reported in previous literature, using a confidence level of 95% and a margin of error of 10%, while allowing for a potential 10% non-response rate [11] (Appendices).

Ethical considerations

The study protocol was reviewed and approved by the Institutional Ethics Committee of Assam Medical College and Hospital (approval number: AMC/EC/PG/5362). All study procedures conformed to the ethical standards of the Declaration of Helsinki (2013 revision).

## Results

A total of 96 patients with SCD were enrolled in the study. The mean age of the participants was 31.00 ± 13.89 years. The age distribution showed that the highest proportion of patients, 32 (33.33%), belonged to the 21-30-year age group, followed by 25 (26.04%) in the 31-40-year group, and 15 (15.63%) below 20 years of age. There was a slight male predominance, with 52 (54.17%) patients being male and 44 (45.83%) female. The majority, 71 (76.04%), of patients belonged to the tea tribe community, reflecting the ethnic predisposition of SCD in this population.

Regarding systemic clinical features, pallor, 85 (88.54%), and generalized weakness, 83 (86.46%), were the most common presenting complaints, followed by painful vaso-occlusive episodes, 51 (53.13%). Splenomegaly was observed in 59 (61.46%) cases, while icterus was noted in 46 (47.92%) patients. These findings are consistent with the known systemic manifestations of chronic hemolytic anemia and episodic vaso-occlusive crises in SCD (Table 1).

**Table 1:** Demographic profile of patients with SCD. SCD = sickle cell disease

Parameter	Number (%)
Total patients	96
Mean age (years)	31.00 ± 13.89
Age group <20	15 (15.63%)
Age group 21–30	32 (33.33%)
Age group 31–40	25 (26.04%)
Age group 41–50	14 (14.58%)
Age group >50	10 (10.42%)
Male (%)	52 (54.17%)
Female (%)	44 (45.83%)
Ethnic group (tea tribe) (%)	71 (76.04%)
Generalized weakness (%)	83 (86.46%)
Pallor (%)	85 (88.54%)
Painful vaso-occlusive episodes (%)	51 (53.13%)
Splenomegaly (%)	59 (61.46%)
Icterus (%)	46 (47.92%)

Evaluation of visual function revealed that approximately one-third of the study population had BCVA between 6/6 and 6/9 in both eyes, 32 (33.33%) right eye, and 33 (34.38%) left eye. A larger proportion exhibited mildly reduced acuity in the range of 6/12 to 6/18, that is, 35 (36.46%) in both eyes. However, a significant subset demonstrated moderate visual impairment, with acuity ≤6/24 observed in 14 (14.58%) (right eye) and 16 (16.67%) (left eye), and ≤6/60 in 14 (14.58%) and 12 (12.50%), respectively (Table 2).

**Table 2:** Visual acuity in both eyes at presentation.

Vision	Right eye		Left eye	
	N	%	N	%
6/6–6/9	32	33.33	33	34.38
6/12–6/18	36	37.50	35	36.46
6/24–6/60	14	14.58	16	16.67
6/60–PL-ve	14	14.58	12	12.50
Total	96	100	96	100

**Figure 1:**
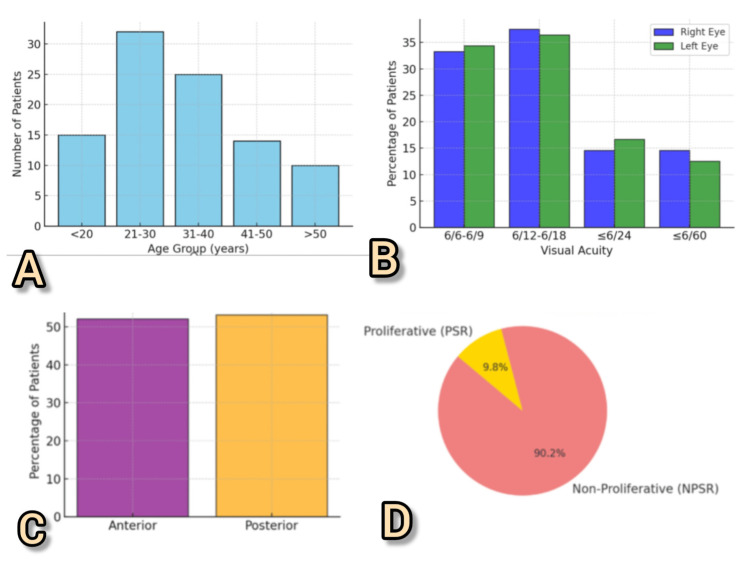
Demographic and clinical ocular profiles in sickle cell disease patients. (A) Age distribution of patients in our study. (B) Visual acuity stratified by eye. (C) Segmental ocular involvement in our patients. (D) Classification of sickle cell retinopathy, showing that most patients had non-proliferative retinopathy. PSR = proliferative sickle retinopathy; NPSR = non-proliferative sickle retinopathy

Anterior segment involvement, predominantly in the form of corkscrew conjunctival vessels, was noted in 50 (52.08%) patients, while posterior segment changes were slightly more prevalent, occurring in 51 (53.13%). Classification of SCR revealed that 46 (47.92%) patients had non-proliferative SCR, whereas five (5.21%) exhibited proliferative changes consistent with neovascularization and advanced retinal ischemia (Figure 1).

**Figure 2:**
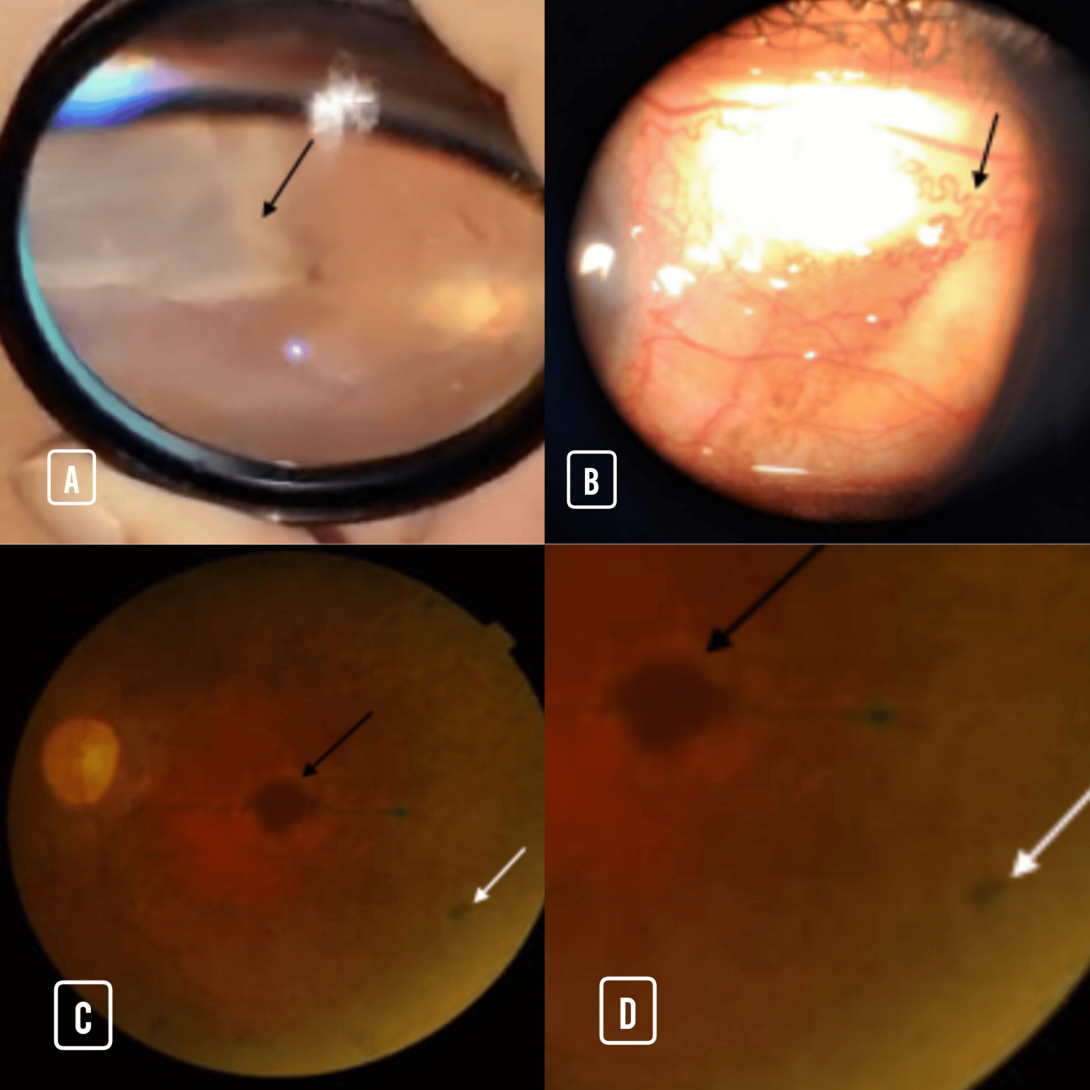
Representative anterior and posterior segment ocular findings in sickle cell disease. A few sickle cell disease patients showed the following findings. (A) Slit-lamp photograph showing corkscrew conjunctival vessels. (B) Indirect ophthalmoscopy image demonstrating retinal detachment. (C) Fundus photograph showing sunburst lesion (black arrow) and chorioretinal scar (white arrow). (D) Enlarged fundus (2×) image confirming similar retinal findings.

On detailed ophthalmological examination, a variety of anterior and posterior segment findings were documented. Slit-lamp biomicroscopy, as illustrated in Figure 2A, revealed prominent corkscrew-shaped conjunctival vessels in several patients, a hallmark of chronic vascular stress and remodeling associated with SCD. Indirect ophthalmoscopy findings included evidence of retinal detachment in a small subset of patients, as demonstrated in Figure 2B, characterized by elevation and undulation of the neurosensory retina. Fundus photography highlighted classic posterior segment lesions consistent with SCR. As seen in Figures 2C, 2D, sunburst lesions, focal areas of retinal pigment epithelial hyperplasia secondary to previous intraretinal hemorrhage, were frequently observed, predominantly in the mid-peripheral retina. Additionally, chorioretinal scars, identified by sharply demarcated hypopigmented areas (white arrows), were present in association with regressed neovascularization or prior ischemic insult. These multimodal imaging findings reinforce the heterogeneity and chronicity of retinal pathology in SCD and underscore the utility of both slit-lamp and retinal imaging in comprehensive ocular assessment.

**Figure 3:**
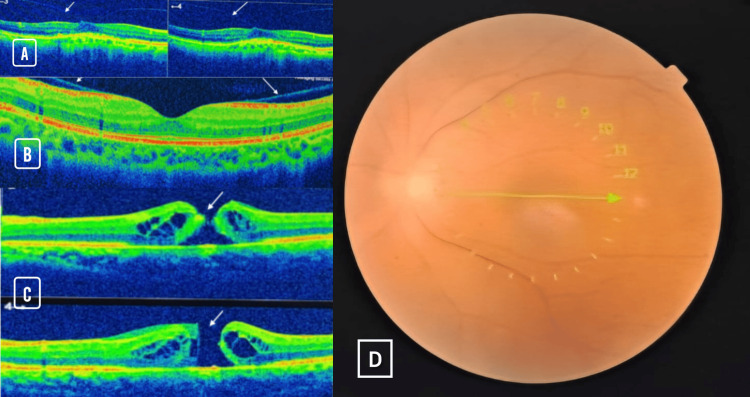
Atypical presentation of spectral-domain OCT findings in macular complications seen in a 36-year-old male with sickle cell disease presenting with weakness, fatigue, and severe anemia. This atypical case shows (A) vitreomacular traction: spectral-domain OCT image showing partial posterior vitreous detachment with persistent adherence of the posterior hyaloid to the fovea, resulting in focal elevation and distortion of the inner retinal contour. The tractional interface is marked by anteroposterior forces leading to subtle inner retinal thickening, without full-thickness disruption. (B) Epiretinal membrane: OCT scan illustrating a hyperreflective line overlying the inner retinal surface, consistent with an epiretinal membrane. The associated surface wrinkling and mild thickening of the inner retina suggest tractional changes secondary to fibrocellular proliferation. The foveal depression appears shallow but remains structurally intact. (C) Full-thickness macular hole: sequential spectral-domain OCT scans demonstrating complete dehiscence of the foveal architecture with a vertical gap extending through all retinal layers, indicative of full-thickness macular holes. Surrounding the hole, intraretinal cystic changes and retinal edema are visible. The operculum is seen detached anteriorly in some cases. These findings reflect chronic tractional and ischemic damage, with potential for significant central visual loss if left untreated. (D) Fundus image of the patient showing a macular hole (arrow). OCT = optical coherence tomography

Spectral-domain OCT provided further insights into macular structural alterations among patients with SCD. Figure 3A demonstrates vitreomacular traction, evidenced by focal elevation of the internal limiting membrane and distortion of the foveal contour due to persistent adherence of the posterior hyaloid. Figure 3B reveals the presence of an epiretinal membrane, visible as a hyperreflective band along the retinal surface, associated with mild wrinkling and underlying retinal thickening, findings indicative of membrane-induced retinal distortion. The most advanced pathology is illustrated in Figure 3C, which shows full-thickness macular holes in two patients, characterized by complete interruption of the neurosensory retina at the fovea, with surrounding cystoid changes and operculum formation. These OCT findings underscore the impact of chronic ischemic and tractional processes in SCD, highlighting the importance of early macular assessment in preventing central vision loss.

**Figure 4:**
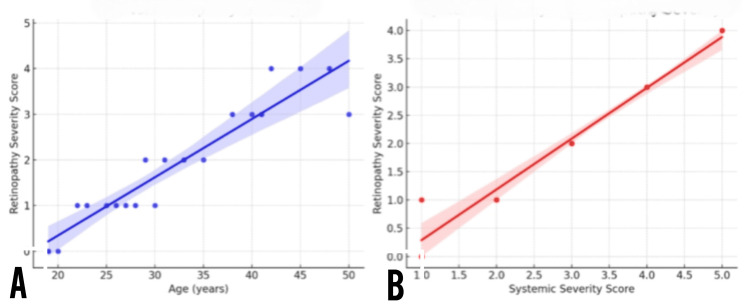
Correlation between retinopathy severity and age/systemic disease in sickle cell disease. (A) Scatter plot showing a positive correlation between patient age and retinopathy severity score, indicating increasing retinal involvement with advancing age. (B) Scatter plot demonstrating a strong linear association between systemic severity score and retinopathy severity, suggesting systemic disease burden as a predictor of ocular complications.

Correlation analysis revealed a significant positive association between patient age and the severity of retinopathy. As depicted in the left panel of Figure 4, increasing age was correlated with higher retinopathy severity scores (R² = 0.76, p < 0.01), suggesting a progressive cumulative burden of retinal microvascular damage over time in individuals with SCD. Similarly, a strong linear relationship was observed between systemic severity scores and the degree of retinopathy, as illustrated in the right panel of Figure 4 (R² = 0.94, p < 0.001). Patients with more severe systemic manifestations, including frequent vaso-occlusive crises, splenomegaly, and hemolytic features, were more likely to exhibit advanced ocular involvement. These findings emphasize the intertwined pathophysiology between systemic and ocular complications in SCD and support the implementation of integrated care models that prioritize early ocular screening in patients with high systemic disease burden.

## Discussion

SCD is a genetically inherited hemoglobinopathy characterized by the substitution of valine for glutamic acid at the sixth position of the β-globin chain, resulting in the production of abnormal HbS. This structural alteration leads to red blood cell sickling, promoting chronic hemolysis and vaso-occlusive episodes. Among its multisystemic complications, ocular involvement, particularly in the form of SCR, is a significant but often underrecognized cause of visual morbidity [1,2].

Epidemiology and risk stratification

The prevalence and severity of SCR vary according to SCD genotype, age, and systemic disease burden. PSR has been reported in up to 43% of individuals with HbSC and 14% of those with HbSS genotypes by the age of 24-26 years [6]. This discrepancy, despite HbSS being the more severe systemic phenotype, underscores the need for genotype-specific ocular surveillance [7]. In the present study, a similar trend was observed, where posterior segment involvement and PSR were disproportionately more prevalent in older patients and those with systemic complications such as vaso-occlusive crises and splenomegaly.

Pathophysiological mechanisms

SCR results from microvascular occlusion caused by rigid, sickled erythrocytes, leading to ischemia and compensatory neovascularization. Goldberg’s classification remains a cornerstone for stratifying PSR progression into the following five stages: peripheral arterial occlusion, arteriovenular anastomosis, neovascular proliferation (sea-fan formation), vitreous hemorrhage, and, ultimately, retinal detachment [3,8]. Progressive retinal ischemia and secondary neovascular responses are particularly evident in mid-peripheral retinal zones, often detected only through wide-field imaging modalities [12].

Clinical spectrum and imaging findings

Ocular involvement in SCD spans both anterior and posterior segments. Anterior segment findings include conjunctival corkscrew vessels, seen in over half of our cohort. Posterior segment manifestations ranged from non-proliferative lesions, such as salmon patch hemorrhages, iridescent spots, and black sunburst lesions, to overt PSR with sea-fan neovascularization and vitreous hemorrhage [9]. Spectral-domain OCT and fluorescein angiography provided crucial insights into macular pathology. OCT images revealed epiretinal membranes, vitreomacular traction, and full-thickness macular holes, changes indicative of chronic ischemic and tractional damage [10,13].

Emerging imaging modalities such as OCT angiography and ultra-widefield fluorescein angiography have further revolutionized early detection. These tools enable the visualization of subclinical ischemic changes and peripheral non-perfusion zones, even in asymptomatic individuals [14,15]. Studies from Egypt and Nigeria corroborate our findings, with reports of macular thinning and high SCR prevalence in pediatric and adult cohorts [8,16].

Therapeutic interventions

Management of SCR depends on disease staging. Asymptomatic non-proliferative stages require periodic monitoring. For PSR, panretinal laser photocoagulation remains the mainstay of treatment, targeting avascular ischemic zones to induce neovascular regression [15]. Anti-vascular endothelial growth factor therapies, such as intravitreal bevacizumab, have shown efficacy in managing active neovascularization, especially in eyes not amenable to laser therapy [17]. Surgical intervention, including pars plana vitrectomy, is indicated for non-resolving vitreous hemorrhage or tractional retinal detachment [18]. These strategies are supported by long-term observational data highlighting improved outcomes with timely intervention [19].

Correlation with systemic disease and prognostic implications

A key finding of this study was the significant correlation between the severity of systemic manifestations and ocular involvement. Patients with higher systemic severity scores, marked by recurrent vaso-occlusive crises, pallor, and splenomegaly, were more likely to exhibit advanced retinopathy. This observation aligns with previous work emphasizing systemic disease control as integral to preserving ocular function [6,7]. Our scatter plot analysis further reinforced age and systemic severity as strong predictors of retinopathy burden.

Strengths and limitations

This study provides valuable insights into the ocular manifestations of SCD, with a particular emphasis on retinal involvement. A key strength lies in the use of comprehensive ophthalmologic evaluation, including advanced imaging modalities such as OCT and FFA, which enabled precise characterization of retinal abnormalities. The inclusion of patients recruited from multiple departments (Ophthalmology, Medicine, and Pediatrics) enhanced the representativeness of the study cohort, while the systematic application of Goldberg’s classification added methodological rigor and clinical relevance. Furthermore, the study highlights important associations between systemic severity and ocular involvement, reinforcing the link between systemic disease control and ocular outcomes.

Nevertheless, several limitations must be acknowledged. The cross-sectional design inherently restricts the ability to draw causal inferences or evaluate the longitudinal progression of retinopathy. The data presented represent associations at a single point in time; hence, causality and temporal relationships between systemic severity and ocular manifestations cannot be established. Additionally, although the study draws attention to genotype-specific differences in retinopathy risk (HbSS vs. HbSC), stratified analysis by genotype was not performed due to a lack of complete genotype data. This limitation should be explicitly noted, as it constrains the ability to fully assess the differential risk profiles across genotypes. Future studies with genotype-stratified cohorts would strengthen these observations.

The relatively modest sample size (n = 96) may also limit the statistical power, particularly for subgroup analyses, and increase the risk of type II error. Another limitation is that only bivariate analyses were performed (e.g., associations of age and systemic severity with retinopathy), without adjustment for potential confounders such as genotype, sex, or duration of disease. Addressing these factors would require a larger sample size and multivariate modeling. Finally, the lack of longitudinal follow-up precludes assessment of disease progression, treatment outcomes, or recurrence of retinopathy after intervention.

Taken together, while the study contributes significantly to the understanding of ocular involvement in SCD within this regional population, future longitudinal, multi-center studies with larger, genotype-stratified cohorts and multivariate analyses are needed to provide a more comprehensive understanding of risk factors and long-term outcomes.

## Conclusions

This study underscores the importance of early and routine ophthalmic screening in patients with SCD, especially those with HbSC genotype or elevated systemic disease activity. The integration of advanced retinal imaging into clinical practice enables the timely detection of sight-threatening complications. Future longitudinal studies with larger, multi-ethnic cohorts are warranted to refine genotype-specific screening protocols and assess long-term outcomes of emerging treatment strategies.

## Figures and Tables

**Figure 5:**
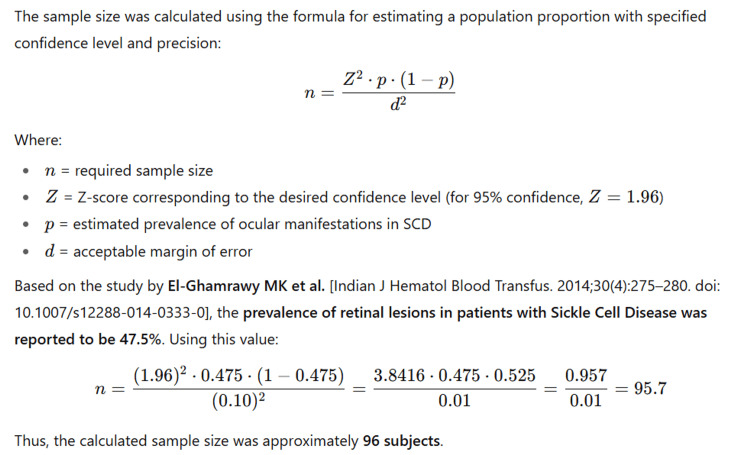
Sample size calculation.
